# Intraprostatic Botulinum Toxin Type A injection in patients with benign prostatic enlargement: duration of the effect of a single treatment

**DOI:** 10.1186/1471-2490-9-9

**Published:** 2009-08-15

**Authors:** João Silva, Rui Pinto, Tiago Carvalho, Francisco Botelho, Pedro Silva, Rui Oliveira, Carlos Silva, Francisco Cruz, Paulo Dinis

**Affiliations:** 1 Department of Urology, Hospital S. João, Porto, Portugal; 2 Faculty of Medicine, Porto, Portugal; 3 IBMC, Institute for Molecular and Cell Biology, Porto, Portugal

## Abstract

**Background:**

Botulinum Toxin Type-A (BoNT/A) intraprostatic injection can induce prostatic involution and improve LUTS and urinary flow in patients with Benign Prostatic Enlargement (BPE). However, the duration of these effects is unknown. The objective of this work was to determine the duration of prostate volume reduction after one single intraprostatic injection of 200U of Botulinum Toxin Type-A.

**Methods:**

This is an extension of a 6 month study in which 21 frail elderly patients with refractory urinary retention and unfit for surgery were submitted to intraprostatic injection of BoNT/A-200U, by ultrasound guided transrectal approach. In spite of frail conditions, eleven patients could be followed during 18 months. Prostate volume, total serum PSA, maximal flow rate (Qmax), residual volume (PVR) and IPSS-QoL scores were determined at 1, 3, 6, 12 and 18 months post-treatment.

**Results:**

Mean prostate volume at baseline, 82 ± 16 ml progressively decreased from month one coming to 49 ± 9,5 ml (p = 0,003) at month six. From this moment on, prostate volume slowly recovered, becoming identical to baseline at 18 months (73 ± 16 ml, p = 0.03). Albeit non significant, serum PSA showed a 25% decrease from baseline to month 6. The 11 patients resumed spontaneous voiding at month one. Mean Qmax was 11,3 ± 1,7 ml/sec and remained unchanged during the follow-up period. PVR ranged from 55 ± 17 to 82 ± 20 ml and IPSS score from10 to 12 points.

**Conclusion:**

Intraprostatic BoNT/A injection is safe and can reduce prostate volume for a period of 18 months. During this time a marked symptomatic improvement can be maintained.

## Background

Botulinum Toxin type A (BoNT/A), the strongest biological neurotoxin known to man, recently has been investigated as a treatment for multiple disorders, including lower urinary tract dysfunction [1].

BoNT/A investigation for the treatment of benign prostatic enlargement (BPE) due to benign prostatic hyperplasia (BPH) started in 2003 [2] after the experimental demonstration that intraprostatic injection of the neurotoxin induced prostatic atrophy in the Rat [3]. In an exploratory study, which involved injection of 200 U of BoNT/A (Botox) in moderate to large prostate glands, a rapid prostate volume decrease was induced and still present at 12 months [2]. Following this study, other groups reported similar findings when injecting prostate glands of 50 ml or larger [4-7]. BoNT/A injection in smaller prostate glands caused a much lesser reduction in prostate volume but a 15% reduction was still observed [8-10]. Independently of the extent of prostate volume reduction, improvement of LUTS and flow were consistently reported [2,4,8,9] while a decrease in total serum PSA were observed in some studies [2].

As stressed in a recent review [11], the duration of prostate atrophy after a single BoNT/A needs to be determined. Unfortunately, all the studies that reported on one single intraprostatic injection of BoNT/A had a limited follow up, during which no signs of prostate re-growth were observed. Recently Brisinda et al [7] reported on repeated neurotoxin injections dictated either by the lack of symptomatic improvement or by symptomatic deterioration. However duration of prostate atrophy after a single injection was again overlooked despite a follow-up of up to 30 months [7].

Quite recently, we had the opportunity to follow 21 frail elderly patients during 6 month after 200U BoNT/A administration. [12]. Eleven of these patients could be followed at regular intervals in our outpatient clinic for a further 12 months. Here we report on the changes in prostate volume observed during the 18 month period. In addition the evaluation of total serum PSA, lower urinary tract symptoms and urinary flow was also investigated.

## Methods

All patients gave written informed consent to participate in this study, which was approved by the Ethics Committee of our Hospital. BoNT/A was offered as a compassion procedure to patients on indwelling catheter and at high risk to undergo prostate surgery due either to cardio-pulmonary co-morbidities or to terminal disease. All patients had been on indwelling urethral catheter for at least 3 months because of urinary retention refractory to several attempts (at least 2 in each patient) of catheter removal under alpha-blocker medication. At the time of enrolment, all patients had stopped alpha blocker and/or 5-alpha-reductase inhibitor medication and none of these compounds were prescribed thereafter. Patients received the standard evaluation for benign prostatic enlargement, which comprised a physical examination including digital rectal examination, prostate volume determination by transrectal ultrasound, kidney and bladder ultrasound, and biochemical blood tests including total prostate-specific antigen (PSA). Patients with a clinical suspicion of harbouring prostate cancer were excluded. Exclusion criteria included total PSA above 6,5 ng/ml or a suspicious digital rectal examination. In one patient with a baseline PSA of 14,5 ng/ml, prostate cancer was excluded after a negative 12 core prostate biopsy.

From the initial group of 21 frail patients followed for 6 months and reported elsewhere (mean age of 80 years) [12], eleven patients managed to attend all their scheduled visits up to 18 months post injection (1, 3, 6, 12 and 18 months). Ten patients from the initial group could not be followed during this time length. Five remained in urinary retention despite BoNT/A injection and received additional treatments that prevented further evaluation of BoNT/A induced prostate volume changes therefore being excluded. Two died from underlying oncologic disease. Three could not attend the outpatient clinic at regular intervals due to a profound deterioration of their general health condition.

The eleven patients, as previously reported [12], received BoNT/A by transrectal approach under transrectal ultrasound guidance. Two vials of BoNT/A, (200 U of neurotoxin, Allergan, Irvine, CA, USA) were diluted in 8 ml of saline. A 21-G, 20-cm long Chiba needle was placed in the adapter of a transrectal endosonic linear transducer (Siemens, Germany) and 4 ml (100 U) were injected in two different places in each transitional zone while the diffusion was controlled by ultrasound. Patients were sent home with a Foley catheter and instructed to take ciprofloxacin 500 mg twice a day for 7 days. One month later a transrectal ultrasound of the prostate was repeated and blood collected for total PSA. The Foley catheter was then removed and all the 11 patients had spontaneous micturition. Maximum flow rate (Qmax) and post void residual volumes (PVR) were determined. Patients maintained spontaneous voiding up to the 18^th ^month visit. At all visits prostate volumes, total PSA, Qmax and PVR were determined. From the 3 month visit on, IPSS and QoL scores were also evaluated (earlier IPSS determination was precluded since patients were on a catheter at baseline and during the first month after BoNT/A). All ultrasound examinations were performed by one single urologist (JS) and the values presented refer to the total gland and not to the transitional zone volume. Results are presented as mean values ± standard error (SE). Mean values of each parameter were compared by a non parametric Wilcoxon Signed Ranks Test for paired data. A p < 0.05 was considered statistically significant.

## Results

The mean age of this sub-cohort was 81,7 ± 2,6 years (range, 61–92). Injection was well tolerated. No patient required analgesic therapy, developed acute prostatitis, haematuria, urethral bleeding or symptoms of botulinum disease.

**Figure 1:**
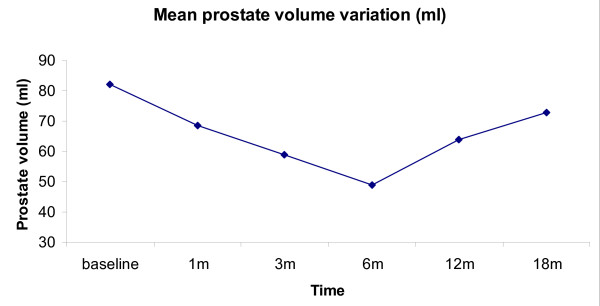
**Mean prostate volume variation**.

Table 1 indicates the mean values of each parameter. Mean prostate volume was 82,2 ± 16 ml at baseline and decreased gradually to a minimum of 49 ± 9,5 ml (p = 0,002) at 6 month, corresponding to a 40% reduction. From this date on a slow re-growth of the glands was observed and at 18 months mean volume was already similar to baseline, 73 ± 16 ml, (p = 0,05). Figure 1 shows mean prostate volume variation from baseline to the 18^th ^month.

**Table 1:** Baseline characteristics and results at 1, 3, 6, 12 and 18-month evaluation. Results were compared to baseline or the first evaluation.

	Baseline	1 month	3 months	6 months	12 months	18 months
Prostate volume	82,2 ± 16,2	68,7 ± 15,4 (p < 0,001)	59,1 ± 10,3 (p = 0,008)	49 ± 9,5 (p = 0,002)	63,8 ± 14 (p = 0,002)	73 ± 16 (p = 0,05)

t PSA	6,7 ± 2,1	6,6 ± 2,4 (p = 0,9)	5,1 ± 1,5 (p = 0,09)	5,1 ± 1,4 (p = 0,16)	5,4 ± 1,3 (p = 0,3)	5,9 ± 1,6 (p = 0,5)

PVR	-	73 ± 18	82 ± 20 (p = 0,7)	55 ± 17 (p = 0,4)	64 ± 16 (p = 0,7)	58 ± 11 (p = 0,5)

Q max	-	11,3 ± 1,7	12,0 ± 1,8 (p = 0,7)	12,3 ± 1,9 (p = 0,5)	11,4 ± 1,4 (p = 0,9)	10,5 ± 1,1 (p = 0,6)

IPSS	-	-	12,3 ± 1	10 ± 0,6 (p = 0,03)	10,8 ± 1,1 (p = 0,2)	11,3 ± 0,4 (p = 0,4)

Qol	-	-	3,3 ± 0,4	2,4 ± 0,2 (p = 0,12)	3 ± 0,1 (p = 0,6)	3,2 ± 0,2 (p = 0,9)

Mean total serum PSA decreased from 6,7 ± 2,1 ng/ml at baseline to 5,07 ± 1,5 ng/ml and 5,09 ± 1,4 ng/ml at 3 and 6 months respectively, corresponding to a 25% reduction. However these changes did not reach statistical significance (p = 0,09 and p = 0,16, respectively) At last observation mean value was 5,9 ± 1,6 ng/ml (p = 0,47). All patients voided *per *urethra after catheter removal at first month follow-up visit, and all maintained spontaneous voiding since then. Mean Qmax was 11,3 ± 1,7 ml/sec at 1^st ^month and maintained similar values along the 18 months of observation (Table 1). IPSS and associated QoL scores were first evaluated when patients came to the 3 month evaluation and were 12,3 ± 1 and 3,3 ± 0,4, respectively. These scores were maintained without significant variations along the follow-up period. Mean post-void residual volume was 73 ± 18 ml at the 1^st ^month and also did not show significant variations during the surveillance period.

## Discussion

The main finding coming from the analysis of this sub-cohort was the duration of prostate atrophy after the single injection of 200U of BoNT/A which was found to be about 18 months. As previously stressed, the duration of this period had never been reported. In addition we found that the nadir of prostate volume occurred 6 months after the neurotoxin administration.

The decrease of prostate volume uniformly described after intraprostatic BoNT/A injections [2,10,12,13] should be related with the widespread apoptosis that has been detected in the gland after BoNT/A administration in rats, dogs, and humans. Other mechanisms such as a straight necrotic effect of the injected solution seems improbable as cavitations were not observed in the gland tissue during transrectal ultrasounds repeatedly carried out in this or in other studies [2-10,12,13]. Apoptosis involves both the epithelial and stromal components [3,8,14] and recent observations suggest a mechanism of action involving the impairment of the autonomic innervation [15]. As a matter of fact, Silva et al, demonstrated in the Rat that gland apoptosis induced by intraprostatic injection of BoNT/A was caused by the lack of a continuous sympathetic drive [15]. BoNT/A-induced apoptosis could be prevented by maintaining the adrenergic stimulation of the gland with phenylephrine, an adrenergic agent, administered subcutaneously [15]. In spite of the rich cholinergic innervation previously reported in the prostate [16] the impairment of the parasympathetic innervation to the prostate atrophy was less remarkable. Cholinergic stimulation could not prevent BoNT/A-induced atrophy [15].

Despite the marked prostate atrophy, total serum PSA had a very moderate decrease, not exceeding 25% at 3–6 months after BoNT/A. The changes in serum PSA after BoNT/A injection are now at the centre of a lively debate. One would expect that a decrease of total serum PSA would accompany prostate tissue decrease, as usually observed after prostatectomy. Moreover, Maria et al [2,13] and Guercini et al [6,13], reported decreases over 50% in serum PSA levels after prostate volume decreases. It should, however, be stressed that other studies, like us, did not find any decrease in total serum PSA levels regardless of a significant prostate atrophy. This is the case of Chuang et al [8,13] and Park et al [10,13] in which changes of total serum PSA were non-significant. Therefore, at this moment it is unclear why total serum PSA and prostate size volume may not walk side by side after BoNT/A injection. Nonetheless difficulties in matching prostate size and PSA variation have also been observed in other studies. Loeb et al [17] stated that prostate volume increases, determined by MRI, and PSA velocity do not correlate. Despite growth rates as high as 10 cc per year, PSA velocity was less than 0.1 ng/ml per year in most men without prostate cancer [17]. Likewise, prostate size and PSA decreases observed after 5α-reductase inhibitors administration never attained similar values, the decrease of serum PSA reaching 50% whereas prostate volume not exceeding 25% [18].

Resumption of spontaneous voidings by the patients included in this group should not be attributed solely to prostate volume reduction, as a decrease in prostate tonus was also reported after BoNT/A administration [19]. Lin et al observed that injection of 200U BoNT/A significantly reduced prostate urethral pressure in response to intravenous adrenergic agents or electric stimulation in dog prostates. Interestingly, prostate tonus, is under sympathetic control [20], the autonomic innervation that is severely impaired by intraprostatic BoNT/A [15].

As all patients were in indwelling catheterization, PVR, IPSS score and Qmax were evaluated only after the successful voiding that occurred when the urethral catheter was removed 1 month after BoNT/A injection. This moment to remove the catheter was chosen empirically as such a study had never been conducted before. Nevertheless we took in consideration that Maria and coworkers [2] had reported a marked reduction in prostate volume and a discernible improvement in peak urinary flow 1 month after BoNT/A injection, suggesting that at this time point beneficial effects of the neurotoxin were already present. Average IPSS score varied between 10 and 12, clearly indicating that LUTS severity remained low [21] once the catheter was removed and spontaneous voidings were resumed. In accordance, a low score of the associated QoL question was found. In what concerns flow, the mean values ranged between 10,5 and 12,3 ml/sec. Although it might be argued that these Qmax values are not exceptionally high, it should be remembered that at baseline all these 11 patients were on chronic indwelling catheterization refractory to several voiding trials. In addition, PVR remained well below 100 ml during follow up indicating that bladder emptying was rather effective.

Intraprostatic BoNT/A injection was carried out transrectally. This route was used for the first time in our initial study [12] and it was preferred due to the experience the authors accumulated while obtaining prostate biopsies oriented by transrectal ultrasound. In addition, our preliminary attempts revealed that this route is more reproducible than the perineal one described by Maria et al [2] and carries less risk of morbidity, namely hematuria, than the transurethral route used by Kuo et al [4]. Transrectal injections were well tolerated as attested in this cohort by the lack of local or systemic side effects. The fact that general anaesthesia was not required for prostate injection was also a clear advantage as it allowed the procedure in the frail elderly patients included in this study, who had a mean age above eighty years and significant comorbidities. All the patients went home immediately after the treatment which was performed as an office procedure.

One should realise that the study has several limitations. The first comes from the fact that the number of patients that could be followed during 18 months is small and represents a sub-cohort of patients that responded better to BoNT/A injection. However the other 10 patients of the initial cohort that could not be followed for the reasons pointed above, all had a marked atrophy at six months evaluation [12]. Therefore the patients reported in the present study do not represent a special subgroup with prostate atrophy but rather a subgroup with better clinical outcome and/or better clinical condition to attend regular visits. Another limitation comes from the fact that no control group exists, making a bias towards prostate atrophy possible, albeit improbable taking in consideration the magnitude of absolute changes. Pressure/flow studies were not carried out, preventing therefore the correct determination of the cause of the refractory urinary retention. However one must understand that in such a group of frail elderly patients with large prostate glands, urinary retention due to bladder prostatic obstruction is the likely diagnosis. Although bladder decompensation is a well known problem in this aged group of patients, a hypotonic bladder should not be the most important reason for the refractory urinary retention present at inclusion. In fact, these patients voided spontaneously after prostate volume/prostate tonus reduction brought on by BoNT/A. The third limitation is the lack of IPSS scores and QOL scores at baseline, as patients did not void spontaneously at inclusion. Thus, IPSS score, Qmax and PVR determined along the study should be interpreted as an indication that the BoNT/A effect persisted also during a long period of time. Finally, it is unclear if the same time-response will occur if small prostate glands are injected with BoNT/A.

## Conclusion

In conclusion, our study shows that large prostate gland atrophy that follows 200 U injection of BoNT/A is transient, volumes returning to baseline levels after 18 months. These observations may have significant impact for the use of BoNTA in the management of BPE due to BPH.

Intraprostatic BoNT/A injection can reduce the volume of large prostate glands for a period of 18 months. Such a long period of action indicates that intraprostatic BoNT/A injections should be further investigated as a new tool for the management of BPE due to BPH.

## Competing interests

FC is consultant for Allergan and Astellas

## Authors' contributions

JS – designed the study, was involved in clinical assessment of patients, analysed the data and wrote the manuscript. RP, TC, FB, PS, RO – selected, treated and followed the patients. CS, PD – treated patients and analysed the data. FC – participated in the design of the study and its coordination, analysed the data and wrote the manuscript. All the authors read and approved the manuscript.

## Pre-publication history

The pre-publication history for this paper can be accessed here:


